# A quasi-experimental analysis comparing antimicrobial usage on COVID-19 and non-COVID-19 wards

**DOI:** 10.1017/ash.2024.417

**Published:** 2024-10-31

**Authors:** Daniel Doyle, Bruce Dalton, Zuying Zhang, Deana Sabuda, Irina Rajakumar, Elissa Rennert-May, Jenine Leal, John M. Conly

**Affiliations:** 1 Department of Medicine, Cumming School of Medicine, University of Calgary and Alberta Health Services, Calgary, AB, Canada; 2 Pharmacy Services, Alberta Health Services, Calgary, AB, Canada; 3 Infection Prevention and Control, Alberta Health Services, AB, Canada; 4 Department of Community Health Sciences, Cumming School of Medicine, University of Calgary, Calgary, AB, Canada; 5 O’Brien Institute of Public Health, University of Calgary, Calgary, AB, Canada; 6 Calvin, Phoebe, and Joan Snyder Institute for Chronic Diseases, University of Calgary and Alberta Health Services, Calgary, AB, Canada; 7 Department of Microbiology, Immunology, and Infectious Diseases, Cumming School of Medicine, University of Calgary, Calgary, AB, Canada; 8 Department of Pathology and Laboratory Medicine, Cumming School of Medicine, University of Calgary and Alberta Health Services, Calgary, AB, Canada

## Abstract

**Objective:**

To describe antimicrobial usage (AMU) trends before and during the coronavirus disease 2019 (COVID-19) pandemic, between COVID-19 and non-COVID-19 wards, and if there was any association with a COVID-19 order set.

**Design:**

Quasi-experimental retrospective interrupted time series analysis of AMU rates with a contemporaneous comparison of COVID-19 versus non-COVID-19 control wards. Analysis using incidence rate ratios (IRR) was conducted using a Poisson regression generalized linear model.

**Setting:**

Five COVID-19 and 4 comparable non-COVID-19 wards and 6 intensive care units (ICUs) at 4 hospitals during pandemic waves 1–4.

**Participants:**

All inpatients receiving systemic antimicrobials.

**Intervention:**

The COVID-19 checkbox antimicrobial order set was implemented in March 2020, to be used only if considered clinically indicated with modification in August 2021.

**Main Outcome(s) and Measure(s):**

The primary outcome was a change in AMU rates (defined daily dose per 100 patient days per month) comparing pre- versus peri-pandemic periods and COVID-19 versus control non-COVID-19 wards. Secondary outcomes included antifungal usage rate in ICUs and assessing AMUs following implementation and modification of a COVID-19 order set.

**Results:**

Significantly greater rates of AMU (IRR[95%CI]) were observed on COVID-19 wards versus non-COVID-19 wards during waves 1–4 for all systemic antimicrobials (1.76[1.71–1.81], 1.10[1.07–1.13], 1.48[1.43–1.53], and 1.06[1.03–1.09]); for azithromycin (11.76[9.80–14.23], 10.96[9.49–12.74], 12.41[10.73–14.45], and 4.88[4.31–5.55]); and for ceftriaxone (2.39[2.16–2.65], 3.64[3.29–4.03], 2.94[2.67–3.23], and 1.62[1.49–1.76]).

**Conclusions:**

We observed significantly increased AMU rates of all systemic agents during the first 4 waves of the pandemic and on COVID-19 wards compared with control wards for azithromycin and ceftriaxone. These agents saw a twofold reduction following order-set removal, suggesting that the clinical decision-support tool order set, as utilized, had influenced prescribing behavior.

## Introduction

Following the coronavirus disease 2019 (COVID-19) pandemic declaration by the World Health Organization (WHO) on March 27, 2020,^1^ 7 waves were observed across Alberta, Canada, up until July 2023.^2^ Little was initially known about this novel coronavirus, resulting in frequent changes to diagnostic, management, and prevention recommendations, with significant jurisdictional variation.^3^ Antimicrobial usage (AMU) has significantly been affected by the COVID-19 pandemic, in both the acute care^4,5^ and outpatient settings.^6^

Antimicrobial resistance (AMR) contributes to increased patient morbidity, mortality, and healthcare costs.^7,8^ The Infectious Diseases Society of America (IDSA) Antimicrobial Stewardship Guidelines describe a strong association between unnecessary AMU and resistance.^8^ A 2022 comprehensive global AMR analysis estimated that in 2019 approximately 5 million deaths were associated with AMR, and over one-quarter of those deaths were directly caused by a resistant organism.^9^ Nearly a quarter of prescriptions in Canadian healthcare facilities were deemed suboptimal or inappropriate between 2018 and 2019.^10^ Expenditures related to AMR by the Canadian healthcare system at that time were estimated at 1.4 billion dollars.^10^

The extent of bacterial co-infections in COVID-19 patients has been an area of uncertainty since the start of the pandemic. This uncertainty has led to variable practices globally with regard to the empiric use of antimicrobials in patients admitted with COVID-19.^3^ Despite the high rates of AMU seen in patients admitted to hospital with COVID-19^11^ and the relatively high burden of AMR in COVID-19 patients reported in a recent systematic review,^12^ the literature suggests relatively low rates of bacterial co-infection. A 2022 systematic review found bacterial co-infection rates and AMU in patients admitted with COVID-19 to be estimated at 5.62% and 61.8%, respectively.^11^ The vast majority of AMU in this population may be unnecessary.

Regarding collateral effects of excess AMU, incidence rates of invasive fungal infections in critically ill patients with COVID-19 have been reported to be increased, ranging from 5% to 26% in the literature. These infections are predominantly associated with *Aspergillus* species, agents of mucormycosis, and *Candida* species.^13^

There is a relative dearth of specific quantitative prescribing trends since the start of the pandemic in Canada. We therefore sought to assess trends in systemic AMU on acute care inpatient wards in a large Canadian metropolitan setting. We compared overall AMU trends before and during the COVID-19 pandemic in adult acute care and intensive care units (ICUs), examining overall AMU and specific antimicrobials used for respiratory infections, and AMU trends on dedicated COVID-19 (henceforth COVID) wards compared with comparable non-COVID control wards. As a secondary objective, we sought to examine antifungal use specifically in the intensive care setting during COVID due to a perceived increased use in this population.

## Methods

### Study setting and period

Our study reviewed AMU data in Calgary Zone, Alberta, Canada, a metropolitan health region in Western Canada serving a population of 1.4 million^14^ with 4 adult acute care hospitals and approximately 2,490 beds. AMU data was collected starting 24 months prior to the pandemic to allow comparison to pre-pandemic AMU trends. The study period ran from April 1, 2018, to December 31, 2021. A total of 15 wards were analyzed including 5 COVID wards (with 1 from each of 3 hospitals and 2 from the largest hospital), 4 non-COVID wards, and 6 ICUs, the latter units having variable numbers of COVID patients at any given time.

Dates for pandemic waves 1 through 4 were determined using COVID data from the Government of Alberta website.^2^ Wave 1 occurred between March and June 2020, wave 2 between October and February 2021, wave 3 between March and June 2021; and wave 4 between August and December 2021.

### Study design

This study used a quasi-experimental design with interrupted time series analysis comparing AMU on a month-to-month basis, using a before-and-after design for the COVID and non-COVID designated wards along with a controlled comparative analysis of COVID wards versus non-COVID wards. Dedicated medical COVID wards were used as test cohorts and comparable non-COVID, and hospitalist general medical wards were used as control cohorts at each site. Where hospitalist medical wards could not be analyzed, general internal medicine wards not designated as COVID wards were used as a substitute control group. These wards were felt to have comparable patient profiles to their COVID ward counterparts, with predominantly cardiorespiratory illnesses, diabetes mellitus, and metabolic disorders. COVID wards were intermittently reassigned for various reasons during the study but were analyzed accordingly to reflect the COVID ward designation at that time whereby a control ward was also switched to maintain a consistent control ward and vice versa.

A locally developed electronic order set was implemented on March 27, 2020, for inpatients at all acute care sites with severe acute respiratory coronavirus virus 2 (SARS-CoV-2) infection. In addition to containing non-antimicrobial orders for SARS-CoV-2 patients, checkboxes were included for both azithromycin and ceftriaxone as potential treatment options for community-acquired pneumonia. Management recommendations were in accordance with adaptations of AMMI Canada^15^ and IDSA guidelines.^16^ Both antimicrobials were removed from the order set on August 26, 2021. The timing of order-set implementation and antimicrobial removal coincided with waves 1 and 4, respectively. Changes in prescribing trends were reviewed overall as well as in the context of the dates corresponding to significant interventions associated with this order set.

AMU was quantified using a defined daily dose (DDD per 100 patient days), as defined by the WHO.^17^ This metric facilitated the comparison of AMU over time, both locally and nationally. Routes of administration included oral, intravenous, and intramuscular. Data from all other routes of administration, outpatient data, and data from patients under the age of 18 were excluded.

### Data collection and sources

Monthly population-level data was extracted from pharmacy electronic medical records similar to the previous methodology.^18^ Data collected included ward, site (COVID versus non-COVID control ward), units of antimicrobial dispensed, ward patient days per month, ward, COVID admission rates per month, and length of stay (LOS) where available. Data was collected for overall systemic antimicrobial therapy, as well as the following specific antimicrobials: azithromycin, ceftriaxone, levofloxacin, meropenem, piperacillin-tazobactam, and vancomycin. Data for doxycycline was included for non-intensive care wards. Data for antifungal use was included for ICUs including overall systemic antifungal use and specific agents including amphotericin B, micafungin, fluconazole, voriconazole, posaconazole, and isavuconazole.

### Ethics

The Alberta Research Ethics Community Consensus Initiative ethics screening tool^19^ was used to assess this study which scored this project as fitting with a quality improvement project for which ethics approval was not required. No individual patient-specific data was retained for this initiative.

### Analysis

Descriptive epidemiologic statistics including mean, standard deviation, median, and interquartile range were calculated to compare DDD per 100 patient days between pandemic waves and the pre-pandemic period. Incidence rate ratios (IRR) and 95% confidence intervals (CI) were calculated by comparing the pre-pandemic period to each of the 4 waves in our analysis. Trending plots were conducted to examine the rates of DDD per 100 patient days and were broken down by year and month. Trends in DDD per 100 patient days were explored using interrupted time series analysis. A generalized linear model with Poisson distribution and a log link function was used. In the Poisson regression model, DDD was used as the response, with the offset specified as the logarithm of patient days, with time in months and the COVID wave as the predictive factor (Appendix Table 14). A *P* value <0.05 was considered statistically significant. All statistical analyses were conducted using R version 4.1.0 statistical software.

## Results

**Figure 1.**
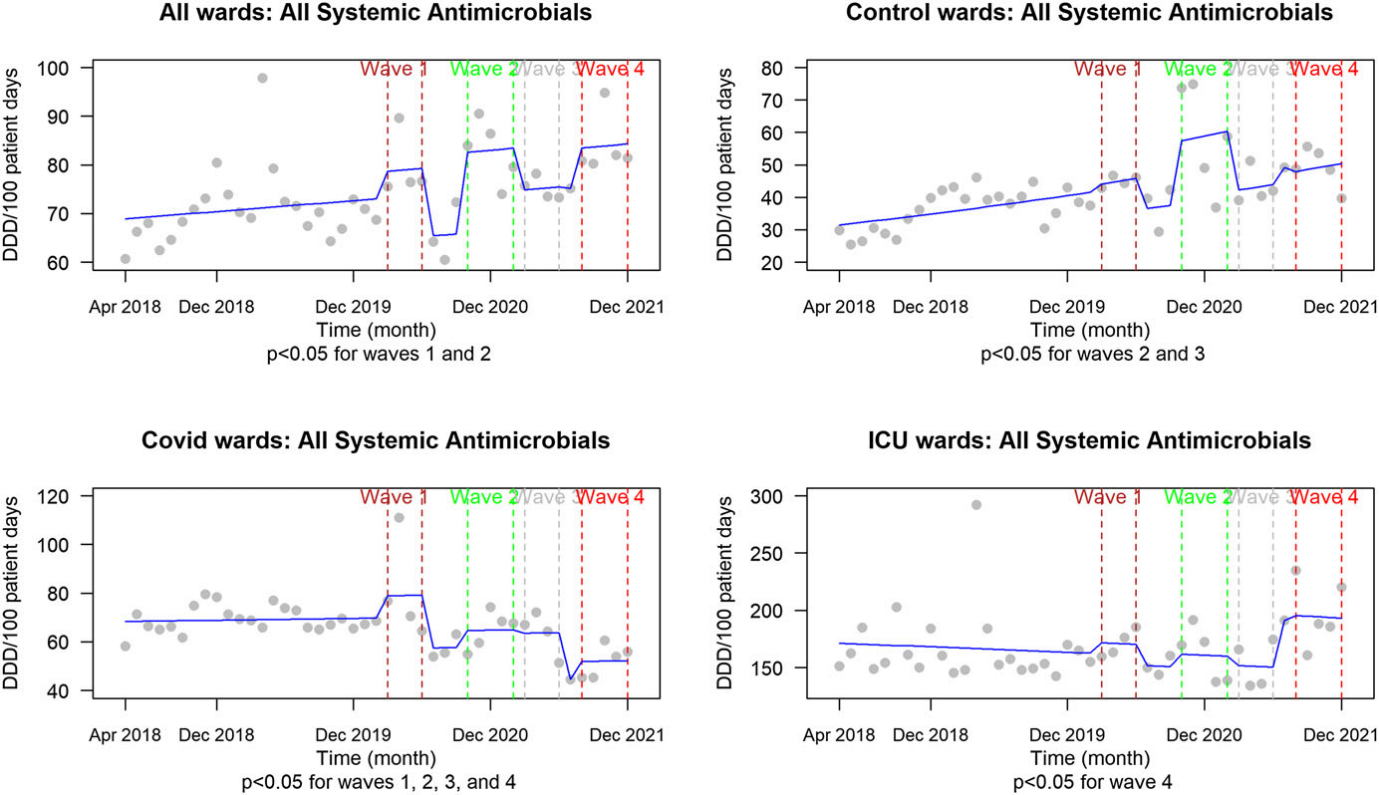
All systemic antimicrobial usage (DDD per 100 patient days) from 2018 to 2021 representing the pre-pandemic period compared with each of the first severe acute respiratory coronavirus virus 2 waves. *P* values are reported beneath each graph for the designated comparison. *Note:* DDD, defined daily dose; ICU, intensive care unit.

**Figure 2.**
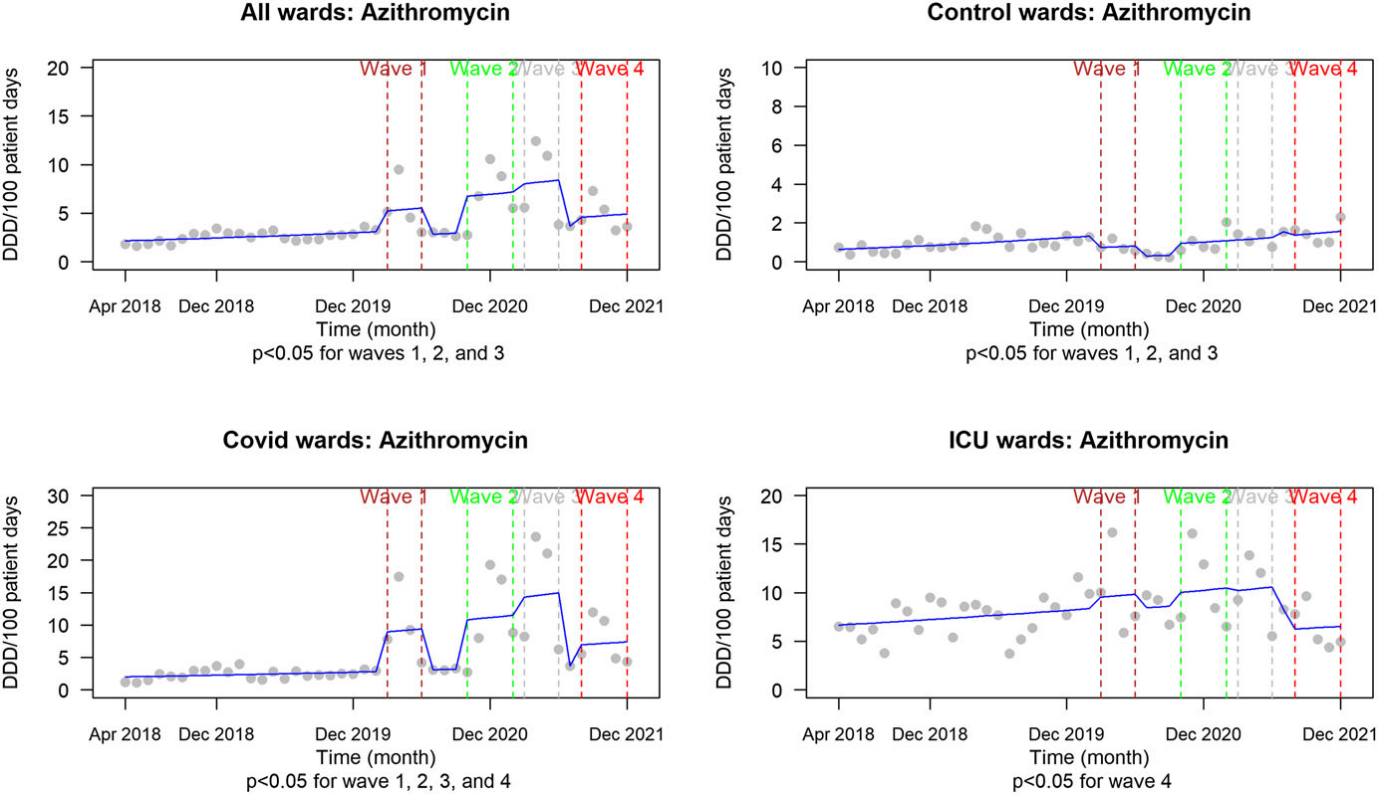
Azithromycin antimicrobial usage (DDD per 100 patient days) from 2018 to 2021 representing the pre-pandemic period compared with each of the first 4 severe acute respiratory coronavirus virus 2 waves. *P* values are reported beneath each graph for the designated comparison. *Note:* DDD, defined daily dose; ICU, intensive care unit.

**Figure 3.**
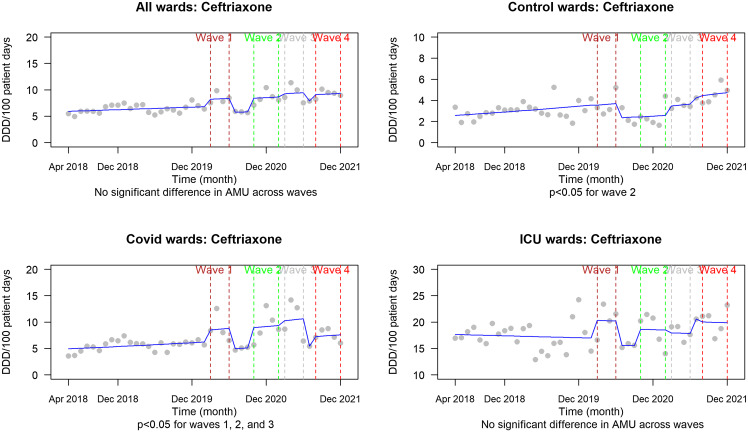
Ceftriaxone AMU (DDD per 100 patient days) from 2018 to 2021 representing the pre-pandemic period compared with each of the first 4 severe acute respiratory coronavirus virus 2 waves. *P* values are reported beneath each graph for the designated comparison. *Note:* AMU, antimicrobial usage; DDD, defined daily dose; ICU, intensive care unit.

We observed significantly increased AMU rates of all systemic agents during the first 4 pandemic waves and on COVID wards versus control wards for azithromycin and ceftriaxone (Figures 1–3).

For doxycycline, there was a significant decrease in rates of use for all wards in waves 3 and 4. There was no significant change in control wards. For COVID wards, there was a significant increase in rates of use seen during wave 1 and a significant decrease observed for all other waves (Appendix Supplementary Data Figure 1a–c). ICU data was not collected.

For levofloxacin, there was a trend toward decreased rates of use for all wards during wave 3, and a significant increase was observed during wave 2. There was no change observed for control wards. COVID wards demonstrated a significant increase in rates of use during wave 1 and a significant decrease during waves 3 and 4 (Appendix Supplementary Data Figure 2a–D).

For meropenem, there was no change in rates of use observed for all wards and a trend toward a decrease in ICU wards during wave 3. There was no change in rates of use observed on control wards. For COVID wards, there was a trend toward an increase in rates of use during wave 1 and a trend toward a decrease during waves 2 and 3 (Appendix Supplementary Data Figure 3a–d).

For piperacillin-tazobactam, there was no change in rates of use observed for all wards. There was a significant decrease in rates of use for both ICU and COVID wards during waves 3 and 4, and a significant decrease was observed in control wards during wave 1 (Appendix Supplementary Data Figure 4a–d).

For parenteral vancomycin, there was no change in rates of use observed for all wards, control wards, or ICU wards. On COVID wards, there was a decrease in rates of use during waves 2, 3, and 4 (Appendix Supplementary Data Figure 5a–d).

Antifungal rates of use did not demonstrate any significant change in ICU wards during any observed wave (Appendix Supplementary Data Figure 6).

IRR for AMU comparing COVID and control wards are outlined in Table 1. Admissions and LOS were comparable (Appendix Supplementary Table 15).

**Table 1.** COVID versus control ward antimicrobial usage by a wave reported as an incidence rate ratio (IRR)

Wave	IRR	Confidence interval (95%)	*P* value
All systemic antimicrobials			
1	1.76	1.71–1.81	<0.0001
2	1.10	1.07–1.13	<0.0001
3	1.48	1.43–1.53	<0.0001
4	1.06	1.03–1.09	0.0001
Azithromycin			
1	11.76	9.80–14.23	<0.0001
2	10.96	9.49–12.74	<0.0001
3	12.41	10.73–14.45	<0.0001
4	4.88	4.31–5.55	<0.0001
Ceftriaxone			
1	2.39	2.16–2.65	<0.0001
2	3.64	3.29–4.03	<0.0001
3	2.94	2.67–3.23	<0.0001
4	1.62	1.49–1.76	<0.0001
Doxycycline			
1	2.56	2.27–2.88	<0.0001
2	1.40	1.26–1.56	<0.0001
3	1.87	1.65–2.11	<0.0001
4	1.08	0.98–1.18	0.13
Levofloxacin			
1	2.69	2.36–3.07	<0.0001
2	1.44	1.28–1.63	<0.0001
3	1.42	1.23–1.64	<0.0001
4	1.05	0.93–1.20	0.4400
Meropenem			
1	3.44	2.62–4.57	<0.0001
2	1.13	0.86–1.48	0.3846
3	0.575	0.44–0.76	0.0001
4	0.606	0.48–0.76	<0.0001
Piperacillin-tazobactam			
1	4.03	3.44–4.74	<0.0001
2	2.35	2.11–2.63	<0.0001
3	1.63	1.43–1.86	<0.0001
4	1.18	1.06–1.31	0.0035
Vancomycin (parenteral)			
1	1.42	1.18–1.72	0.0003
2	1.02	0.84–1.24	0.8215
3	2.39	1.88–3.04	<0.0001
4	1.79	1.43–2.25	<0.0001

## Discussion

Our quasi-experimental study included both before-and-after group analysis as well as a contemporaneous controlled comparative analysis of COVID versus non-COVID wards. One of this study’s objectives was to assess the potential impact of AMU for azithromycin and ceftriaxone prescribing as it pertained to the timing of the COVID admission order set implemented locally in Calgary. These agents were also frequently employed antimicrobials in other studies.^4,5^

There was a significantly increased rate of use seen following the implementation of the order set, concurrent with the start of the pandemic, for both antimicrobials. Aside from the azithromycin data for COVID units, this trend was not observed following the removal of both agents from the set during wave 4 in the time series analysis. The greater azithromycin use observed is likely multifactorial, including the order set, improved outcomes reported early in the pandemic based on observational data, and potentially its anti-inflammatory properties. It is also noteworthy that although COVID unit azithromycin use remained higher than baseline during wave 4, it was lower than the preceding 3 waves. Although there were consistently significant increased rates of use for both antimicrobials on COVID wards compared with control wards across all waves, the magnitude of difference was less following order-set modification, decreasing by approximately twofold for both agents. There are several potential explanations as to why this increase occurred. Early in the pandemic, there was uncertainty around the incidence of concurrent bacterial infections, and clinicians likely prescribed antimicrobials out of an abundance of caution, not wanting to undertreat and potentially cause harm to the patient, in the event there was an incipient bacterial co-infection. Even as more information became available on the low incidence of bacterial co-infection,^11^ the lack of effective treatment options for COVID may have contributed to higher antimicrobial prescribing rates, in addition to the prescribing inertia with antimicrobial agents empirically. It is possible that the inclusion of antimicrobials in the order set led to prescribers feeling justified in including antimicrobials for patients requiring admission to hospital for COVID.

Although the relationship is not causal, this trend of prescribing in the before-and-after analysis does illustrate the potential impact such order sets can have on prescribing behaviors,^20^ and the importance of considering the implications of all additions made to future order sets. Other studies have demonstrated a decrease in AMU later in the pandemic,^21^ which is comparable to our results.

Other Canadian literature on AMU demonstrated an increase in respiratory AMU during wave 1 only on medical wards, as well as an increase in ICU AMU during waves 1 and 2 before returning to baseline during wave 3.^4^ Our comprehensive study saw a common theme in increasing overall AMU on all wards early in the pandemic before returning to pre-pandemic levels, as well as a significant decrease in overall rates of use on COVID wards after the first wave. The exception to this was seen in azithromycin and ceftriaxone rates of use, as previously discussed. We did not specifically look at respiratory antimicrobials combined, so it is an imperfect comparison, although similar patterns exist when comparing our study to related literature.^4^ Another noteworthy exception was seen in ICUs during wave 4 where a significantly increased rate of AMU was observed. This may have been partially driven by variant B.1.617.2 (Delta variant). This variant became the predominant strain of SARS-CoV-2 in Canada during wave 4 of the pandemic^22^ and has been identified as a more virulent strain of SARS-CoV-2, causing more serious illness.^23^

Our study also assessed AMU differences between COVID and control wards. It is interesting that for most respiratory antimicrobials, there was a consistent finding of highly significant increased rates of use on COVID wards for all waves assessed, although the magnitude of difference decreased by wave 4. Exceptions to this trend were observed with meropenem only, where greater rates of use were observed on COVID wards for wave 1 only, with no difference seen in wave 2, and greater use observed on control wards during waves 3 and 4. It may be that patients admitted to non-COVID wards more commonly had different infectious processes than their COVID-infected counterparts requiring broader spectrum antimicrobials.

There is strong evidence of increasing invasive fungal infections in critically ill patients with COVID pneumonia.^13^ For instance, for COVID-associated pulmonary aspergillosis, the prevalence of this condition in critically ill patients was found to be 10% in one review and associated with a mortality rate approaching 60%, with unclear benefit of anti-mold therapy.^24^ COVID-associated mucormycosis, another invasive mold infection, has been shown to have a 50-fold pooled prevalence increase globally, with a mortality rate approaching 30%.^25^

Despite these findings, our study did not demonstrate a change in antifungal use. It may be that our study was underpowered to identify a significant difference, given invasive fungal infections, despite higher incidence being described, remain a relatively uncommon diagnosis. High baseline antifungal use may also contribute to challenges in detecting a significant change, although a retrospective review by Bienvenu et al demonstrated comparable pre-pandemic ICU antifungal usage trends in Calgary.^26^

Strengths of our study include a large data set, comprehensive study design, and complete capture of all AMU data for COVID waves included in our study. All acute care sites admitting adult patients across our health region were included. Of all quasi-experimental designs, the methodology employed has a higher internal validity.^27^ The inclusion of both a control group and pretest group strengthens the epidemiologic associations.^27^ Finally, ours is one of the first studies of which we are aware of comparing COVID and control ward AMU, with longitudinal assessments of AMU prior to and during the pandemic, which has not been commonly presented in the literature to date.

Our study was a single health region in a high-income setting^28^ and as such may limit generalizability to other populations. In addition, due to uncertainty around the management of COVID early in the pandemic, it would have been challenging to assess usage prospectively or in a randomized fashion given the role for empiric antimicrobials was initially unclear. Despite the limitations of our study design, we attempted to mitigate the limitations by including both pretest and control groups.

Our data was not corrected for seasonal variation which can affect AMU. Although COVID incidence may fluctuate, it is relatively seasonally indiscriminate compared with other respiratory viruses.^29^ We felt this made potential confounding due to seasonality less likely.

The use of ward-level data rather than patient-level data is another potential limitation. While pharmacy records are a convenient means of obtaining a large volume of AMU data, this data is not perfect and can overrepresent AMU. For instance, an antimicrobial may have been ordered but not administered for various reasons (loss of IV access, patient refusal). Pharmacy data does not capture these events, as the antimicrobial was dispensed and therefore counted toward overall usage. Unfortunately, looking at individual patient medication administration records was not feasible given the large data set analyzed in this study. Additionally, we did not have detailed length of stay data for individual patients which can contribute to AMU. DDD per 100 patient days is an accepted measurement of incidence density to allow for comparison of antibiotic consumption.

Additionally, we evaluated various parameters including monthly dates for order-set implementation and the pandemic waves. As the exact dates do not perfectly coincide with the beginning and end of respective months, there is the possibility the data analysis for a given month may not be fully representative of the parameter being evaluated. We chose this means of evaluation as the pharmacy AMU data used in the study was provided in monthly intervals. Fortunately, the start and end dates for the parameters listed above did coincide very closely with the beginning and end of each month, which greatly reduces the likelihood of a significant impact on our analysis.

Our study demonstrated an increase in inpatient AMU assessed by DDD per 100 patient days early in the pandemic, as well as a significant decrease following the removal of antimicrobials from the Calgary COVID order set. We demonstrated a temporal association exists between order-set design and prescribing practices and the importance of considering how healthcare decision-support tool content can alter behavior. Our findings suggest that antimicrobial stewardship programs and initiatives should target decision-support tools to optimize AMU both as part of pandemic planning and outside of it. Finally, we believe our study provides a valuable addition to the COVID AMU literature. While our study cannot prove causation, our results clearly demonstrate how prescribing practices have shifted, both as SARS-CoV-2 and our understanding of it evolved over time.

## Supporting information

Doyle et al. supplementary material Doyle et al. supplementary material

## Supplementary material

For supplementary material accompanying this paper visit http://doi.org/10.1017/ash.2024.417. click here to view supplementary material
