# Supplementary material for: A quasi-experimental analysis comparing antimicrobial usage on COVID-19 and non-COVID-19 wards
Source: Antimicrob Steward Healthc Epidemiol. 2024 Oct 31;4(1):e192. doi: 10.1017/ash.2024.417 (PMC11526180; doi:10.1017/ash.2024.417)
Supplement: Doyle et al. supplementary material [file S2732494X24004170sup001.docx]

## **Supplemental Appendix**

Table 1. Mean and median rate of AMU (DDD/100 patient days) for all systemic antimicrobials during the pre-pandemic period and pandemic waves 1-4

| **All Systemic Antimicrobials** | | | | | |
| --- | --- | --- | --- | --- | --- |
| **Ward Type** | **Pre-Pandemic** | **Wave 1** | **Wave 2** | **Wave 3** | **Wave 4** |
| **All** | | | | | |
| Mean (SD) | 99.73 (66.54) | 112.80 (79.03) | 101.55 (58.32) | 94.18 (54.27) | 112.26 (92.72) |
| Median (IQR) | 81.41 (97.11) | 93.03 (97.72) | 79.71 (89.90) | 76.55 (84.07) | 73.37 (120.53) |
| **Control** | | | | | |
| Mean (SD) | 36.84 (11.62) | 46.19 (14.88) | 55.52 (33.51) | 42.32 (13.59) | 46.85 (22.92) |
| Median (IQR) | 34.66 (17.44) | 44.51 (18.93) | 45.04 (15.50) | 39.75 (11.61) | 38.02 (35.38) |
| **Covid** | | | | | |
| Mean (SD) | 70.93 (22.23) | 87.31 (31.48) | 67.95 (14.68) | 66.78 (16.95) | 55.50 (18.00) |
| Median (IQR) | 70.86 (29.39) | 87.61 (37.11) | 68.66 (26.20) | 68.08 (19.95) | 52.78 (20.80) |
| **ICU** | | | | | |
| Mean (SD) | 164.42 (53.96) | 178.43 (83.13) | 160.22 (42.08) | 151.60 (35.12) | 203.16 (83.94) |
| Median (IQR) | 152.72 (48.60) | 163.06 (45.64) | 151.23 (42.20) | 152.31 (47.36) | 179.50 (51.37) |

Table 2. Mean and median rate of AMU (DDD/100 patient days) for azithromycin during the pre-pandemic period and pandemic waves 1-4

| **Azithromycin** | | | | | |
| --- | --- | --- | --- | --- | --- |
| **Ward Type** | **Pre-Pandemic** | **Wave 1** | **Wave 2** | **Wave 3** | **Wave 4** |
| **All** | | | | | |
| Mean (SD) | 4.11 (5.04) | 8.24 (10.09) | 8.54 (8.78) | 9.46 (9.90) | 5.65 (5.41) |
| Median (IQR) | 2.10 (4.93) | 4.15 (10.12) | 5.96 (12.15) | 7.44 (12.65) | 4.12 (7.41) |
| **Control** | | | | | |
| Mean (SD) | 0.96 (0.79) | 0.86 (0.97) | 1.04 (0.93) | 1.11 (1.27) | 1.30 (1.76) |
| Median (IQR) | 0.74 (0.84) | 0.58 (0.91) | 0.91 (0.61) | 0.66 (1.26) | 0.65 (1.00) |
| **Covid** | | | | | |
| Mean (SD) | 2.78 (3.47) | 12.21 (11.35) | 13.50 (10.24) | 16.10 (12.50) | 8.39 (6.55) |
| Median (IQR) | 2.04 (3.87) | 8.54 (15.42) | 13.08 (13.11) | 13.43 (16.73) | 7.59 (7.65) |
| **ICU** | | | | | |
| Mean (SD) | 7.25 (5.89) | 9.85 (9.99) | 9.41 (6.96) | 9.49 (5.86) | 6.27 (4.15) |
| Median (IQR) | 6.07 (9.08) | 7.26 (14.53) | 8.12 (8.79) | 9.96 (9.11) | 5.85 (5.00) |

Table 3. Mean and median rate of AMU (DDD/100 patient days) for ceftriaxone during the pre-pandemic period and pandemic waves 1-4

| **Ceftriaxone** | | | | | |
| --- | --- | --- | --- | --- | --- |
| **Ward Type** | **Pre-Pandemic** | **Wave 1** | **Wave 2** | **Wave 3** | **Wave 4** |
| **All** | | | | | |
| Mean (SD) | 9.54 (8.17) | 12.32 (10.25) | 10.94 (8.14) | 11.42 (7.81) | 11.51 (8.64) |
| Median (IQR) | 6.75 (13.02) | 9.81 (16.15) | 10.63 (11.90) | 11.02 (11.72) | 8.61 (10.72) |
| **Control** | | | | | |
| Mean (SD) | 3.05 (1.54) | 3.74 (2.13) | 2.47 (1.73) | 3.42 (2.25) | 4.43 (2.26) |
| Median (IQR) | 2.95 (1.87) | 3.17 (2.34) | 2.10 (1.24) | 2.83 (2.82) | 4.35 (4.13) |
| **Covid** | | | | | |
| Mean (SD) | 5.82 (4.02) | 10.25 (6.62) | 10.15 (5.10) | 11.15 (6.49) | 8.47 (4.97) |
| Median (IQR) | 5.21 (6.04) | 10.24 (10.33) | 10.63 (7.90) | 10.52 (7.09) | 7.70 (6.32) |
| **ICU** | | | | | |
| Mean (SD) | 16.80 (7.61) | 19.76 (10.84) | 17.24 (7.38) | 16.97 (6.45) | 18.77 (8.35) |
| Median (IQR) | 17.34 (9.30) | 20.59 (16.16) | 15.05 (8.37) | 15.69 (8.37) | 17.99 (12.55) |

Table 4. Mean and median rate of AMU (DDD/100 patient days) for all systemic antifungals during the pre-pandemic period and pandemic waves 1-4

| **All Systemic Antifungals** | | | | | |
| --- | --- | --- | --- | --- | --- |
| **Ward Type** | **Pre-Pandemic** | **Wave 1** | **Wave 2** | **Wave 3** | **Wave 4** |
| **ICU** | | | | | |
| Mean (SD) | 13.96 (9.25) | 15.81 (12.45) | 14.47 (9.58) | 14.82 (9.38) | 13.84 (7.71) |
| Median (IQR) | 12.29 (12.46) | 15.35 (10.72) | 14.87 (11.14) | 12.74 (15.19) | 13.88 (9.54) |

Table 5A. Comparison of all systemic antimicrobial AMU (DDD/100 patient days) during waves 1-4 to the pre-pandemic period (Incidence rate ratio (IRR), 95% Confidence Interval) for all wards


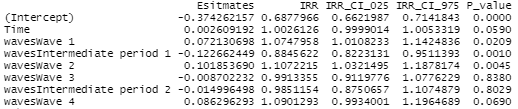


Table 5B. Comparison of all systemic antimicrobial AMU (DDD/100 patient days) during waves 1-4 to the pre-pandemic period (Incidence rate ratio (IRR), 95% Confidence Interval) for control wards


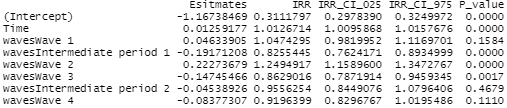


Table 5C. Comparison of all systemic antimicrobial AMU (DDD/100 patient days) during waves 1-4 to the pre-pandemic period (Incidence rate ratio (IRR), 95% Confidence Interval) for Covid wards


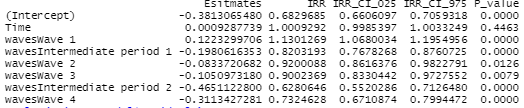


Table 5D. Comparison of all systemic antimicrobial AMU (DDD/100 patient days) during waves 1-4 to the pre-pandemic period (Incidence rate ratio (IRR), 95% Confidence Interval) for ICU wards


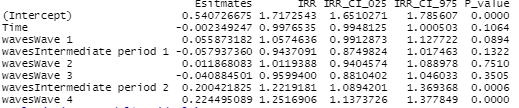


Table 6A. Comparison of azithromycin AMU (DDD/100 patient days) during waves 1-4 to the pre-pandemic period (Incidence rate ratio (IRR), 95% Confidence Interval) for all wards


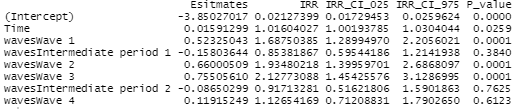


Table 6B. Comparison of azithromycin AMU (DDD/100 patient days) during waves 1-4 to the pre-pandemic period (Incidence rate ratio (IRR), 95% Confidence Interval) for control wards


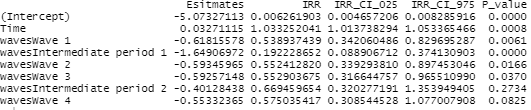


Table 6C. Comparison of azithromycin AMU (DDD/100 patient days) during waves 1-4 to the pre-pandemic period (Incidence rate ratio (IRR), 95% Confidence Interval) for Covid wards


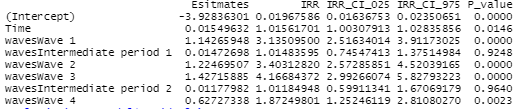


Table 6D. Comparison of azithromycin AMU (DDD/100 patient days) during waves 1-4 to the pre-pandemic period (Incidence rate ratio (IRR), 95% Confidence Interval) for ICU wards


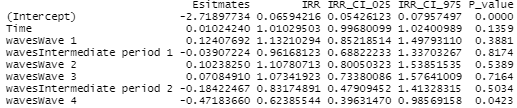


Table 7A. Comparison of ceftriaxone AMU (DDD/100 patient days) during waves 1-4 to the pre-pandemic period (Incidence rate ratio (IRR), 95% Confidence Interval) for all wards


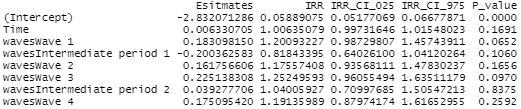


Table 7B. Comparison of ceftriaxone AMU (DDD/100 patient days) during waves 1-4 to the pre-pandemic period (Incidence rate ratio (IRR), 95% Confidence Interval) for control wards


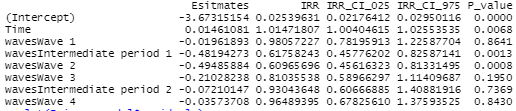


Table 7C. Comparison of ceftriaxone AMU (DDD/100 patient days) during waves 1-4 to the pre-pandemic period (Incidence rate ratio (IRR), 95% Confidence Interval) for Covid wards


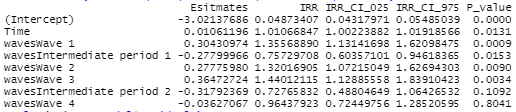


Table 7D. Comparison of ceftriaxone AMU (DDD/100 patient days) during waves 1-4 to the pre-pandemic period (Incidence rate ratio (IRR), 95% Confidence Interval) for ICU wards


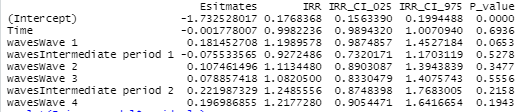


Table 8A. Comparison of doxycycline AMU (DDD/100 patient days) during waves 1-4 to the pre-pandemic period (Incidence rate ratio (IRR), 95% Confidence Interval) for all wards


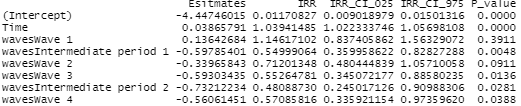


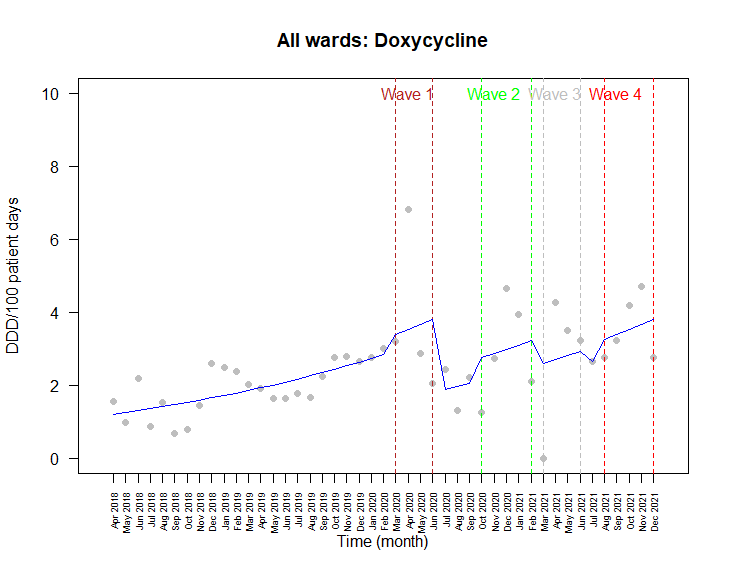


Figure 1A. AMU (DDD/100 patient days) for doxycycline from April 2018 to December 2021 for all wards

Table 8B. Comparison of doxycycline AMU (DDD/100 patient days) during waves 1-4 to the pre-pandemic period (Incidence rate ratio (IRR), 95% Confidence Interval) for control wards


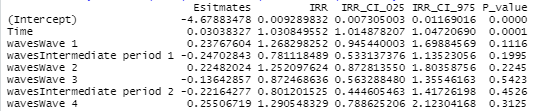


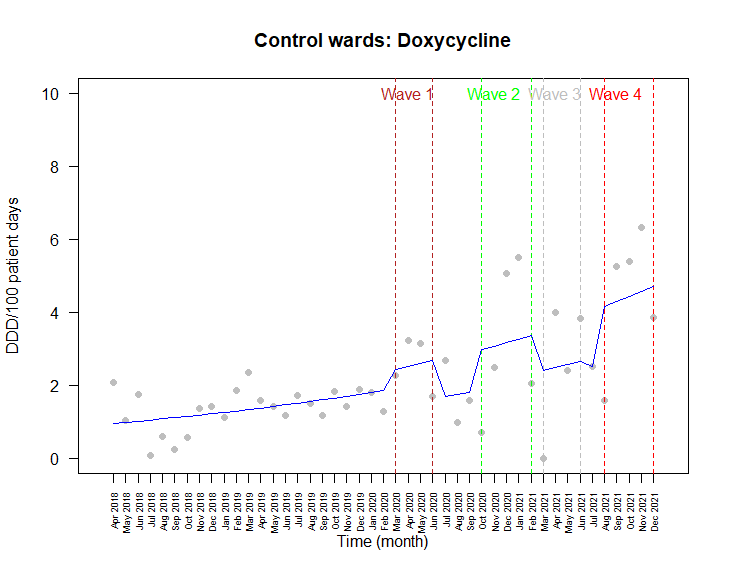


Figure 1B. AMU (DDD/100 patient days) for doxycycline from April 2018 to December 2021 for control wards

Table 8C. Comparison of doxycycline AMU (DDD/100 patient days) during waves 1-4 to the pre-pandemic period (Incidence rate ratio (IRR), 95% Confidence Interval) for Covid wards


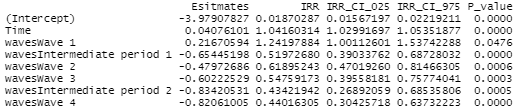


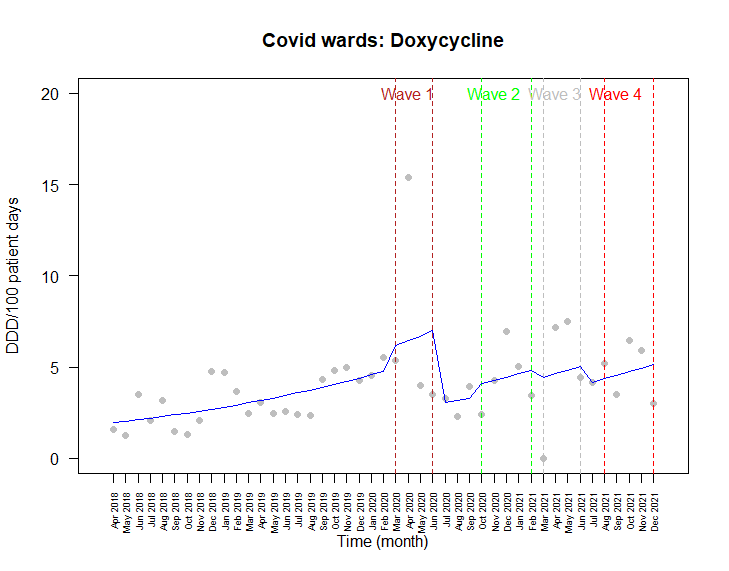


Figure 1C. AMU (DDD/100 patient days) for doxycycline from April 2018 to December 2021 for Covid wards

Table 9A. Comparison of levofloxacin AMU (DDD/100 patient days) during waves 1-4 to the pre-pandemic period (Incidence rate ratio (IRR), 95% Confidence Interval) for all wards


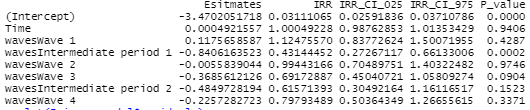


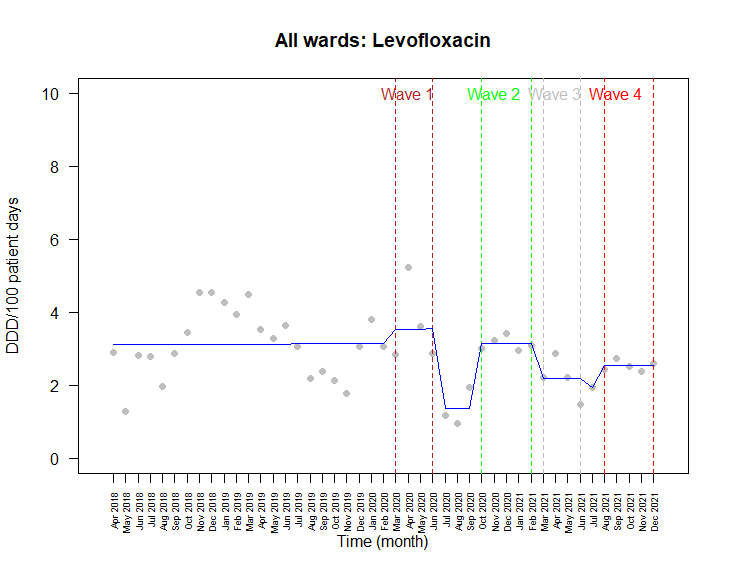


Figure 2A. AMU (DDD/100 patient days) for levofloxacin from April 2018 to December 2021 for all wards

Table 9B. Comparison of levofloxacin AMU (DDD/100 patient days) during waves 1-4 to the pre-pandemic period (Incidence rate ratio (IRR), 95% Confidence Interval) for control wards


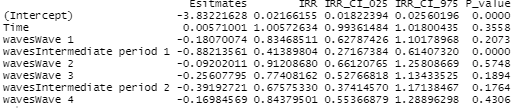


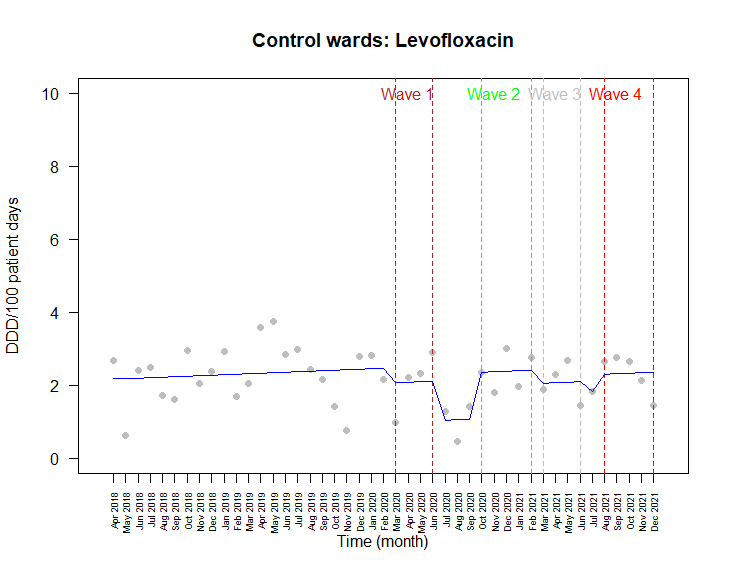


Figure 2B. AMU (DDD/100 patient days) for levofloxacin from April 2018 to December 2021 for control wards

Table 9C. Comparison of levofloxacin AMU (DDD/100 patient days) during waves 1-4 to the pre-pandemic period (Incidence rate ratio (IRR), 95% Confidence Interval) for Covid wards


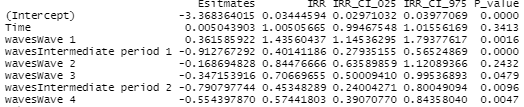


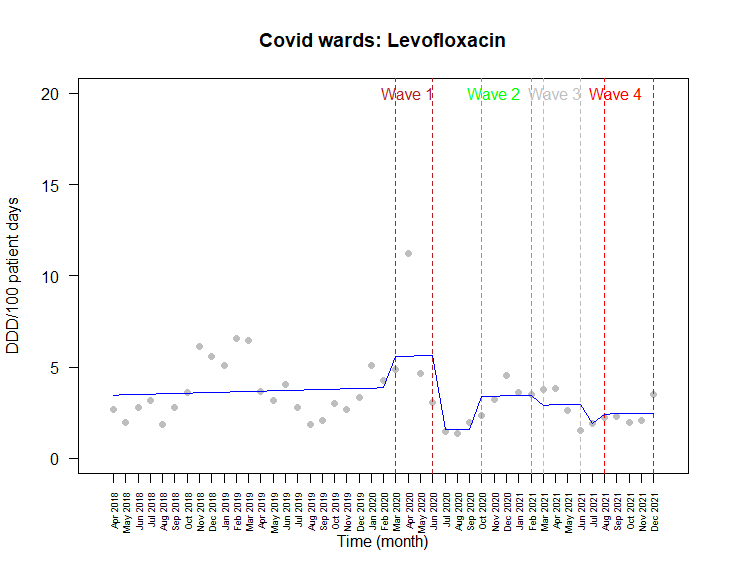


Figure 2C. AMU (DDD/100 patient days) for levofloxacin from April 2018 to December 2021 for Covid wards

Table 9D. Comparison of levofloxacin AMU (DDD/100 patient days) during waves 1-4 to the pre-pandemic period (Incidence rate ratio (IRR), 95% Confidence Interval) for ICU wards


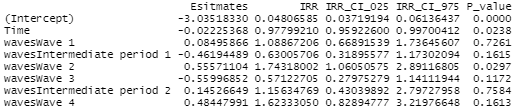


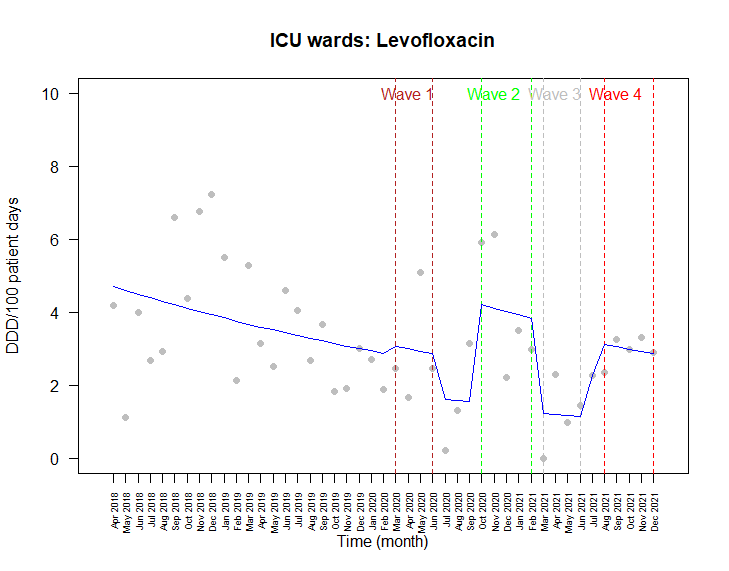


Figure 2D. AMU (DDD/100 patient days) for levofloxacin from April 2018 to December 2021 for ICU wards

Table 10A. Comparison of meropenem AMU (DDD/100 patient days) during waves 1-4 to the pre-pandemic period (Incidence rate ratio (IRR), 95% Confidence Interval) for all wards


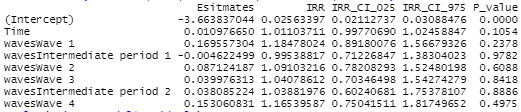


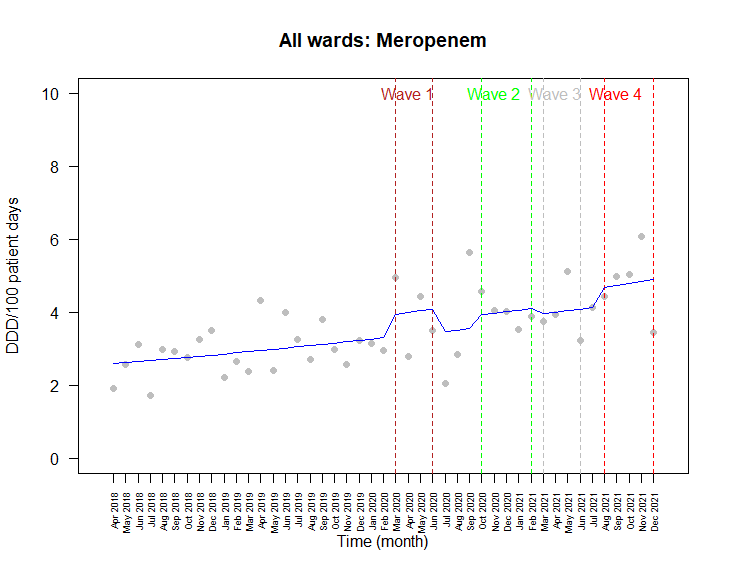


Figure 3A. AMU (DDD/100 patient days) for meropenem from April 2018 to December 2021 for all wards

Table 10B. Comparison of meropenem AMU (DDD/100 patient days) during waves 1-4 to the pre-pandemic period (Incidence rate ratio (IRR), 95% Confidence Interval) for control wards


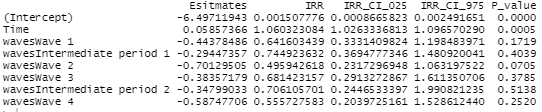


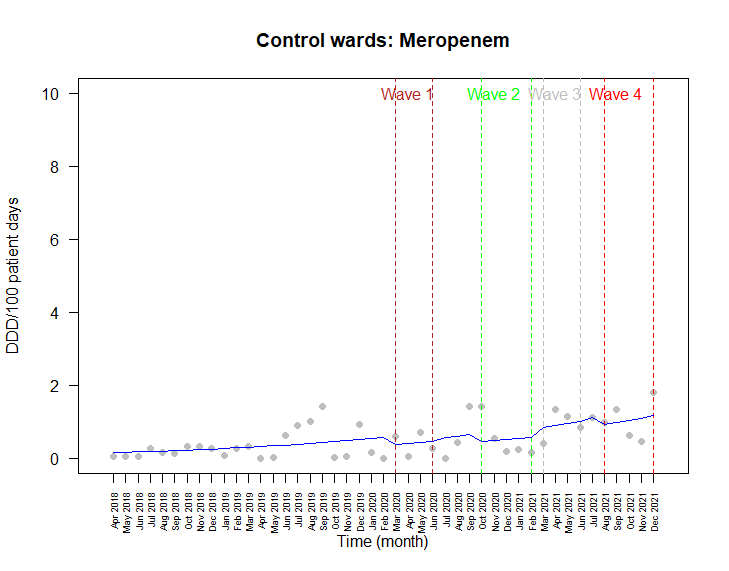


Figure 3B. AMU (DDD/100 patient days) for meropenem from April 2018 to December 2021 for control wards

Table 10C. Comparison of meropenem AMU (DDD/100 patient days) during waves 1-4 to the pre-pandemic period (Incidence rate ratio (IRR), 95% Confidence Interval) for Covid wards


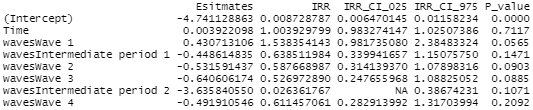


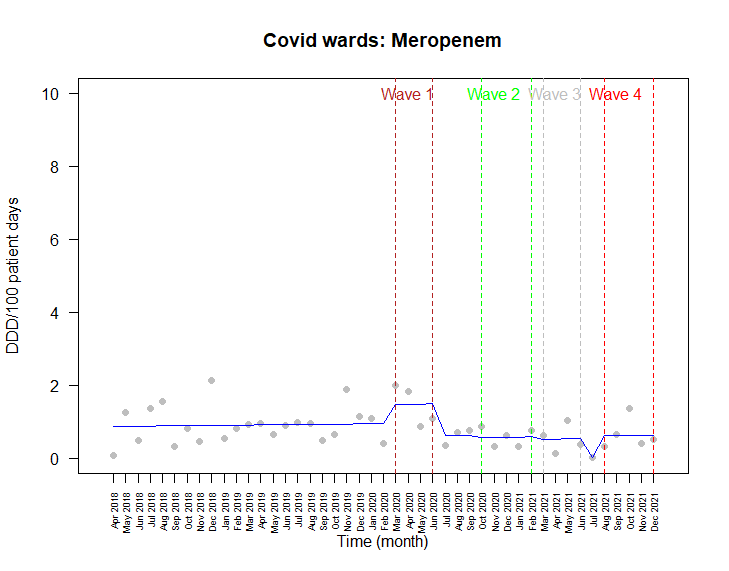


Figure 3C. AMU (DDD/100 patient days) for meropenem from April 2018 to December 2021 for Covid wards

Table 10D. Comparison of meropenem AMU (DDD/100 patient days) during waves 1-4 to the pre-pandemic period (Incidence rate ratio (IRR), 95% Confidence Interval) for ICU wards


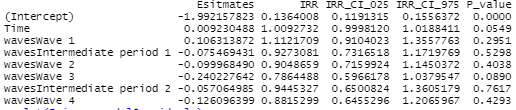


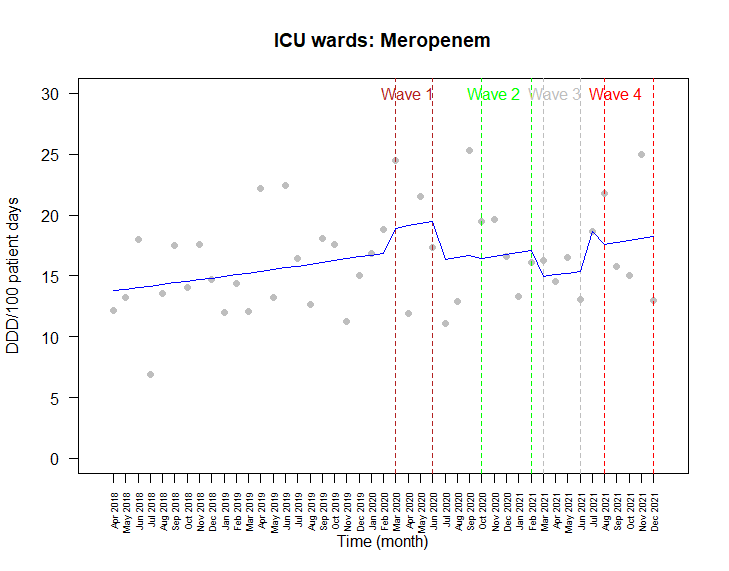


Figure 3D. AMU (DDD/100 patient days) for meropenem from April 2018 to December 2021 for ICU wards

Table 11A. Comparison of piperacillin-tazobactam AMU (DDD/100 patient days) during waves 1-4 to the pre-pandemic period (Incidence rate ratio (IRR), 95% Confidence Interval) for all wards


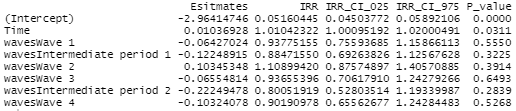


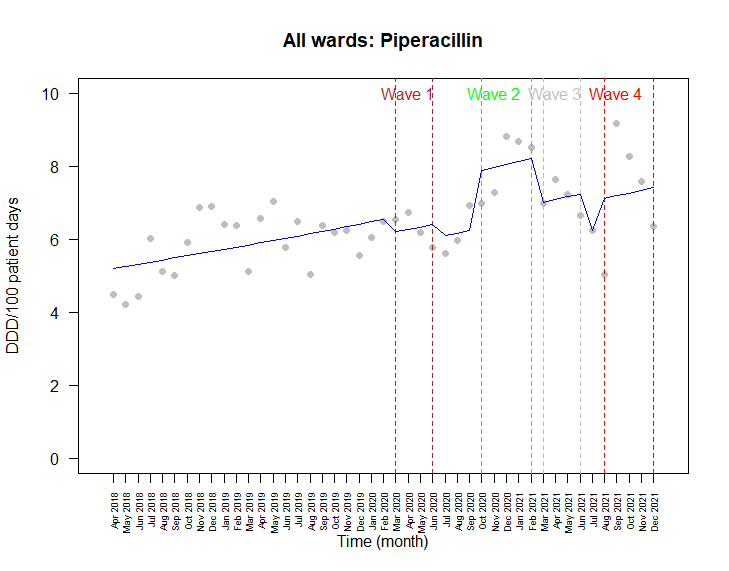


Figure 4A. AMU (DDD/100 patient days) for piperacillin-tazobactam from April 2018 to December 2021 for all wards

Table 11B. Comparison of piperacillin-tazobactam AMU (DDD/100 patient days) during waves 1-4 to the pre-pandemic period (Incidence rate ratio (IRR), 95% Confidence Interval) for control wards


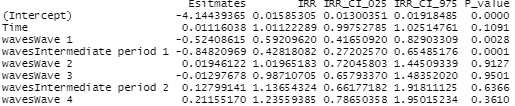


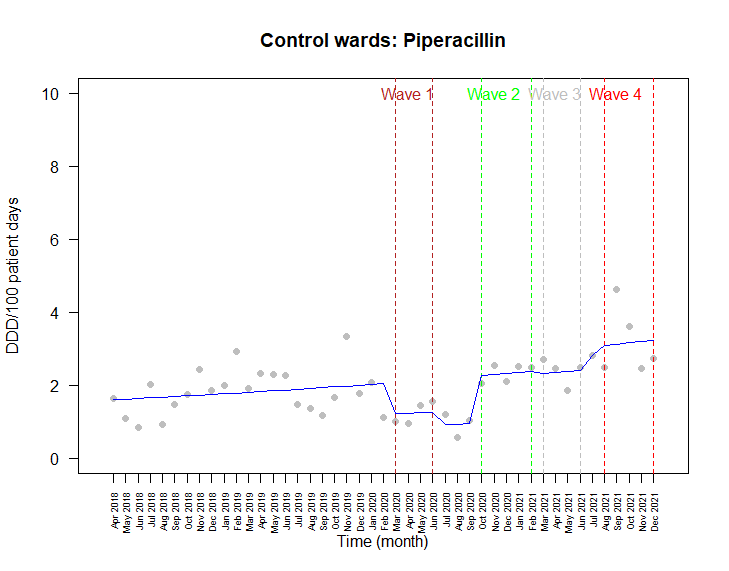


Figure 4B. AMU (DDD/100 patient days) for piperacillin-tazobactam from April 2018 to December 2021 for control wards

Table 11C. Comparison of piperacillin-tazobactam AMU (DDD/100 patient days) during waves 1-4 to the pre-pandemic period (Incidence rate ratio (IRR), 95% Confidence Interval) for Covid wards


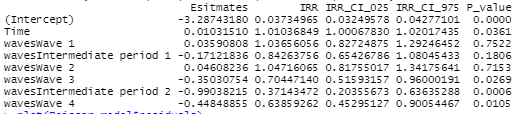


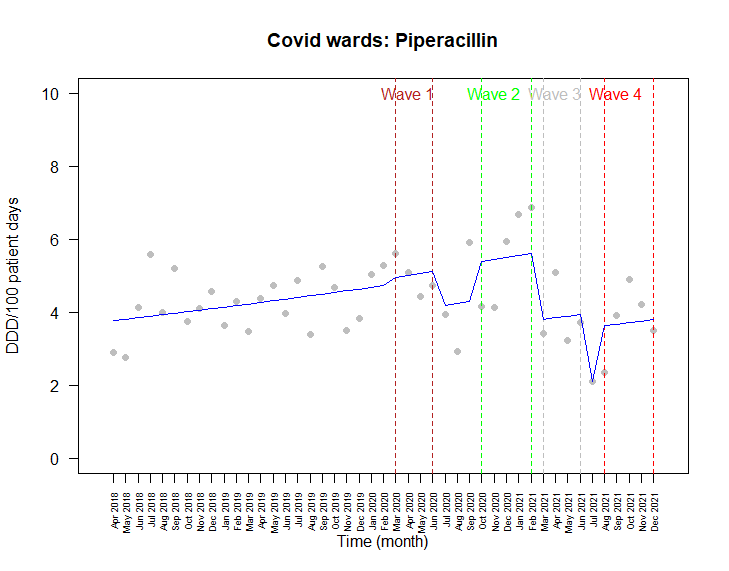


Figure 4C. AMU (DDD/100 patient days) for piperacillin-tazobactam from April 2018 to December 2021 for Covid wards

Table 11D. Comparison of piperacillin-tazobactam AMU (DDD/100 patient days) during waves 1-4 to the pre-pandemic period (Incidence rate ratio (IRR), 95% Confidence Interval) for ICU wards


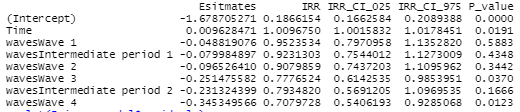


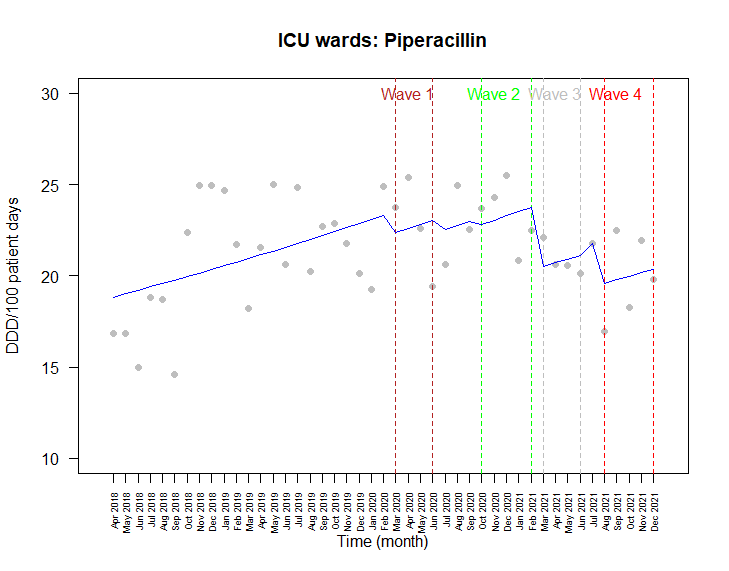


Figure 4D. AMU (DDD/100 patient days) for piperacillin-tazobactam from April 2018 to December 2021 for ICU wards

Table 12A. Comparison of parenteral vancomycin AMU (DDD/100 patient days) during waves 1-4 to the pre-pandemic period (Incidence rate ratio (IRR), 95% Confidence Interval) for all wards


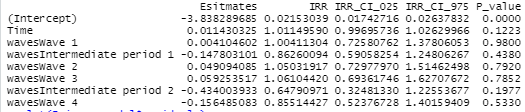


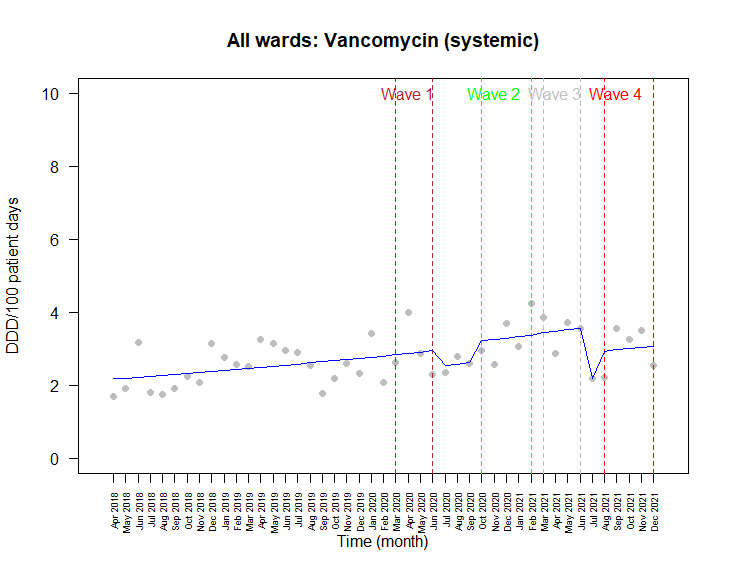


Figure 5A. AMU (DDD/100 patient days) for parenteral vancomycin from April 2018 to December 2021 for all wards

Table 12B. Comparison of parenteral vancomycin AMU (DDD/100 patient days) during waves 1-4 to the pre-pandemic period (Incidence rate ratio (IRR), 95% Confidence Interval) for control wards


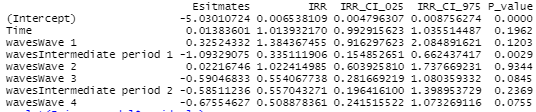


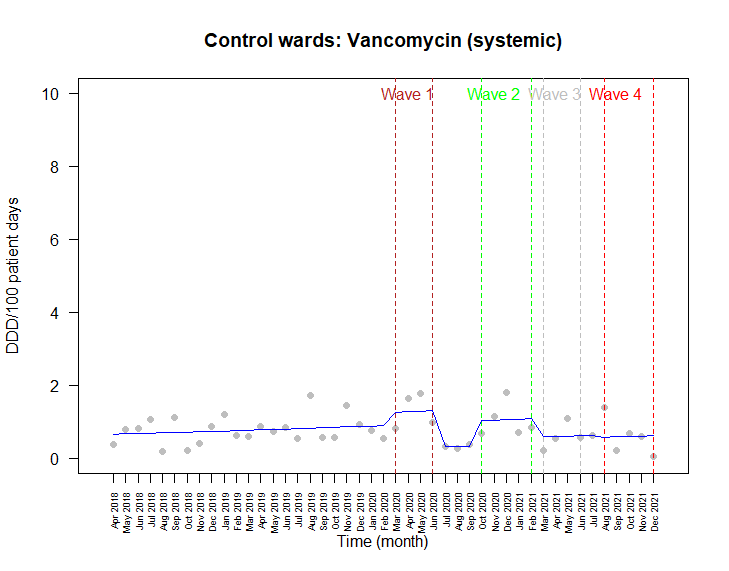


Figure 5B. AMU (DDD/100 patient days) for parenteral vancomycin from April 2018 to December 2021 for control wards

Table 12C. Comparison of parenteral vancomycin AMU (DDD/100 patient days) during waves 1-4 to the pre-pandemic period (Incidence rate ratio (IRR), 95% Confidence Interval) for Covid wards


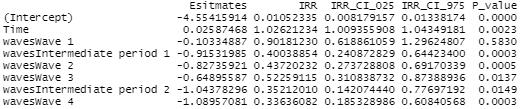


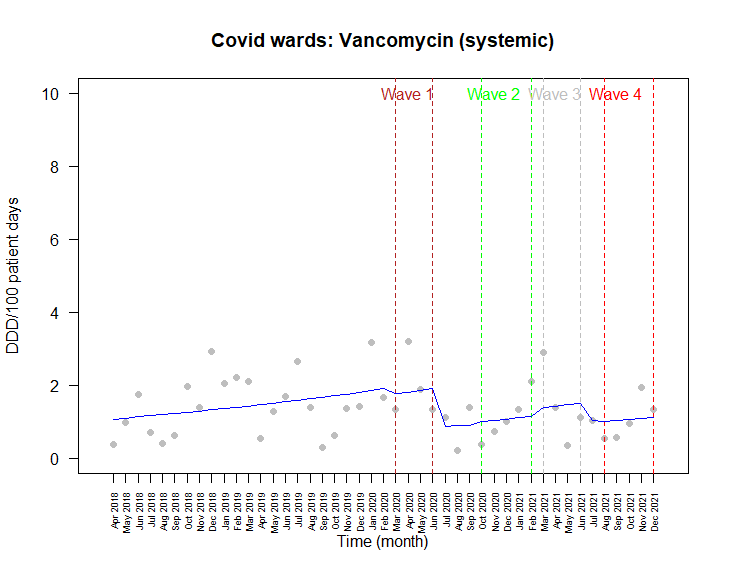


Figure 5C. AMU (DDD/100 patient days) for parenteral vancomycin from April 2018 to December 2021 for Covid wards

Table 12D. Comparison of parenteral vancomycin AMU (DDD/100 patient days) during waves 1-4 to the pre-pandemic period (Incidence rate ratio (IRR), 95% Confidence Interval) for ICU wards


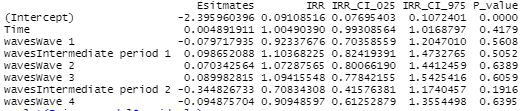


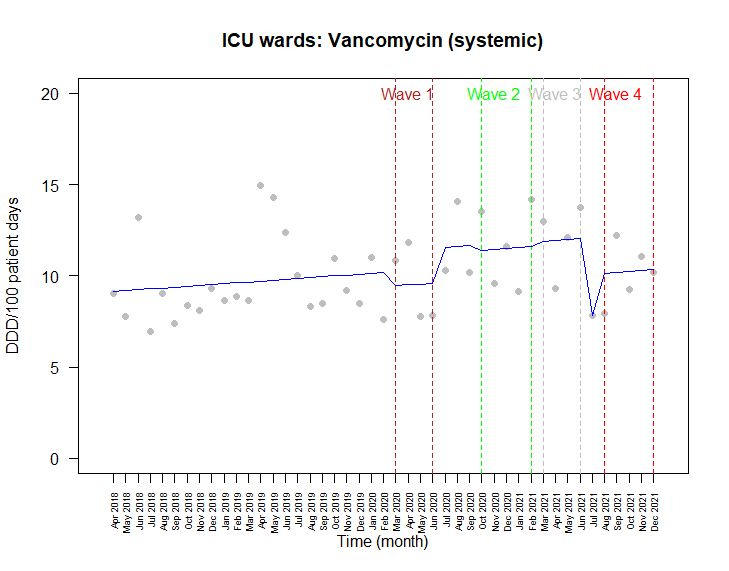


Figure 5D. AMU (DDD/100 patient days) for parenteral vancomycin from April 2018 to December 2021 for ICU wards

Table 13. Comparison of antifungal use (DDD/100 patient days) during waves 1-4 to the pre-pandemic period (Incidence rate ratio (IRR), 95% Confidence Interval) for ICU wards


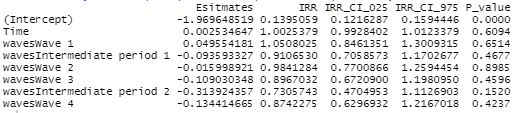


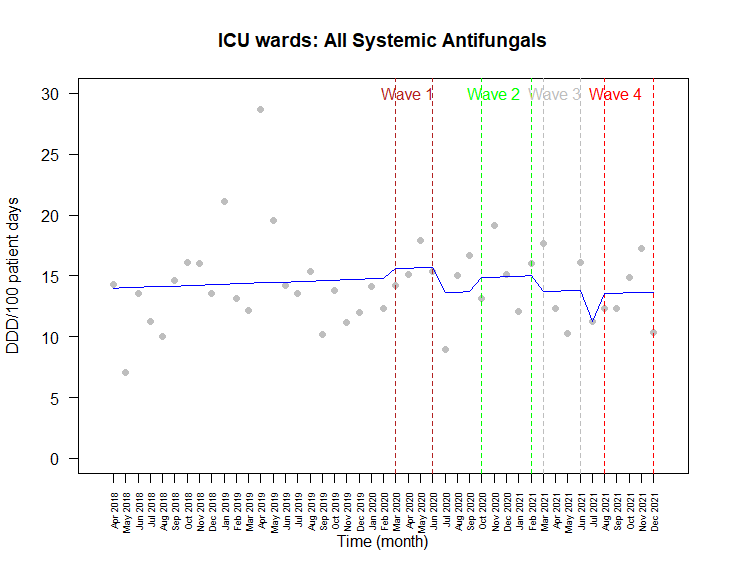


Figure 6. Antifungal rate of use (DDD/100 patient days) for from April 2018 to December 2021 for all wards

| **Time Segment** | **Month / Year** | **Waves** |
| --- | --- | --- |
| 1-23 | April 2018 - February 2020 | Pre-pandemic |
| 24-27 | March - June 2020 | Wave 1 |
| 28-30 | July - September 2020 | Intermediate Period 1 |
| 31-35 | October 2020 - February 2021 | Wave 2 |
| 36-39 | March 2021 - June 2021 | Wave 3 |
| 40 | July 2021 | Intermediate Period 2 |
| 41-45 | August - December 2021 | Wave 4 |

Table 14. Poisson model using a waves variable to differentiate time segments (by month)

Table 15. Length of stay (days) between control and COVID wards at acute care sites March 2020 - December 2021

| **Hospital** | **Ward Type** | **Length of Stay (days)** | **Standard Deviation** |
| --- | --- | --- | --- |
| FMC | Control | 9.5 | 3.5 |
|  | COVID 1 | 9.75 | 8.98 |
|  | COVID 2 | 7.6 | 1.1 |
| PLC† | Control | 7.45 | 1.8 |
|  | COVID | 4.0 | 1.56 |
| RGH* | Control | 7.6 | 1.4 |
|  | COVID | 4.6 | 1.85 |
| SHC | Control | 11.3 | 4.8 |
|  | COVID | 12.0 | 3.8 |

*p<0.001;† p< 0.003 by t test
